# Complete ureteral stenosis after ureteroscopic lithotripsy successfully managed using a simultaneous retrograde and antegrade flexible ureteroscopic approach

**DOI:** 10.1002/ccr3.3507

**Published:** 2020-11-11

**Authors:** Toshitaka Miyai, Takashi Kawahara, Shinnosuke Kuroda, Masato Yasui, Hiroji Uemura

**Affiliations:** ^1^ Departments of Urology and Renal Transplantation Yokohama City University Medical Center Yokohama Japan

**Keywords:** antegrade approach, bilateral ureteroscopic approach, Ho:YAG laser, single‐use flexible ureteroscopy, ureteroscopic lithotripsy

## Abstract

Both retrograde and antegrade approach contributed to the success of a delicate endoscopic procedure. Even when guidewire did not pass thorough to the stenosis lesion, lightning and contrast reagents contributed the way through to the stenosis lesion.

## Graphical abstract

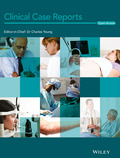

## 1 INTRODUCTION

A 47 year‐old man was referred to our department for the management of ureteral stenosis. Although endoscopic surgery would be difficult, we attempted endoscopic surgery to treat ureteral stenosis with Ho:YAG laser incision. Both retrograde and antegrade approach contributed to the success of a delicate endoscopic procedure.

Postoperative ureteral stenosis is sometimes seen after ureteroscopic lithotripsy due to a mucous membrane injury occurs during lithotripsy or the presence of a residual submucosal stone.^1^ In these cases, endoscopic dilation or uretero‐uretero anastomosis is chosen. In cases of long length ureteral stenosis or severe ureteral adhesion, both procedures are difficult.^2^ We herein report a case of complete ureteral stenosis that was successfully treated using holmium: yttrium aluminum garnet (Ho: YAG) laser incision with a simultaneous retrograde and antegrade ureteroscopic approach.

## 2 CASE PRESENTATION

**FIGURE 1:**
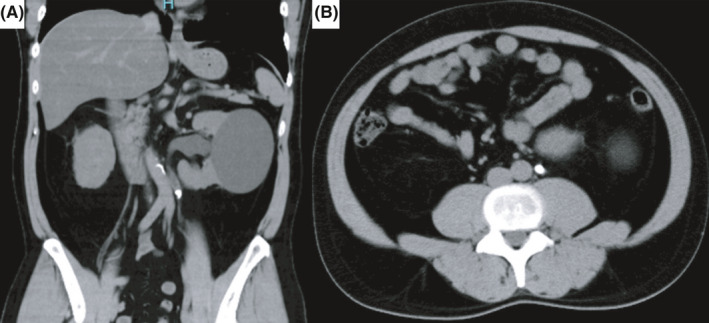
A, Coronal and (B) axial CT images of initial ureteral stone

**FIGURE 2:**
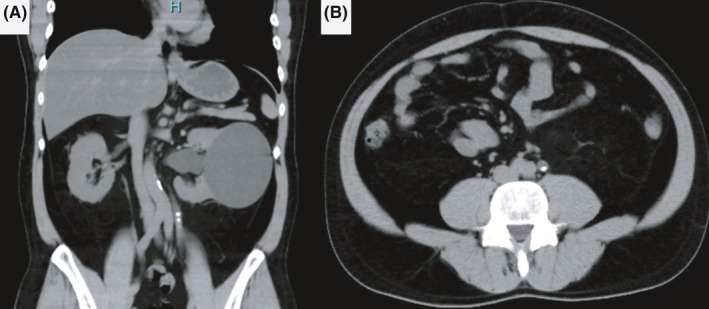
A, Coronal and (B) axial CT images after initial lithotripsy

**FIGURE 3:**
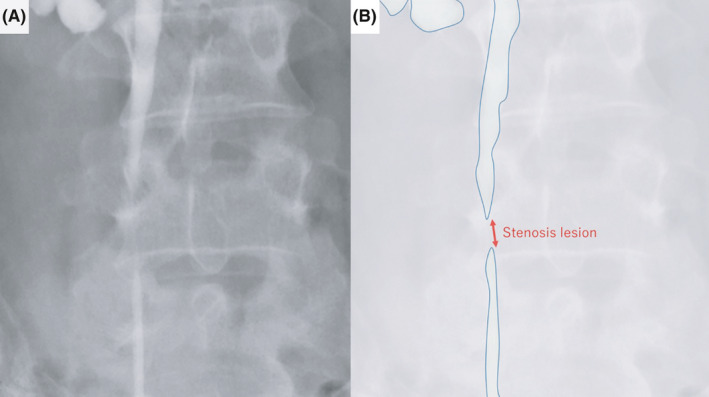
A, Image and (B) schema of ureteral stenosis using bilateral pyelography

The patient was a 47‐year‐old man who had undergone ureteroscopic lithotripsy for a left ureteral stone of 11 mm in size (Figure 1). At the time of the initial treatment, although a residual stone was present, ureteroscopic lithotripsy was ended due to a ureteral mucosal injury and was concluded with the insertion of a double J ureteral stent. Postoperative CT showed a 2‐mm‐diameter residual stone; however, the double J stent was removed (Figure 2). During follow‐up, left hydronephrosis occurred. Retrograde ureteral stenting was attempted but the ureteral stenosis was so severe that contrast reagent did not pass through the upper ureter; thus, a left percutaneous nephrostomy was made. The stenosis lesion was observed around 2 cm (Figure 3).

Four months after initial treatment, the patient was referred to our department for the management of complete left ureteral stenosis. When contrast reagent was injected, it did not pass the stenotic lesion which is 13.5 cm away from uretero‐vesicle junction. Although we considered that endoscopic surgery would be difficult, we attempted endoscopic surgery to treat his ureteral stenosis using a simultaneous retrograde and antegrade ureteroscopic approach with Ho: YAG laser incision.

**FIGURE 4:**
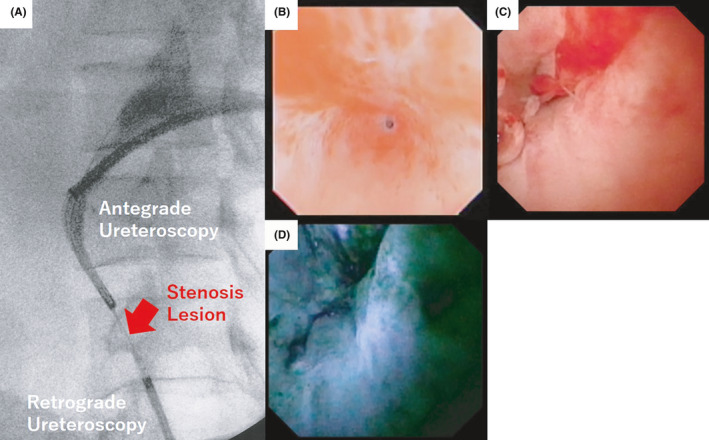
A, Ureteral stenosis lesion at the time of surgery, (B) stenosis lesion from retrograde ureteroscopy, (C) stenosis lesion from antegrade ureteroscopy, and (D) indigo carmine from retrograde ureteroscopy was observed by antegrade ureteroscopy

**FIGURE 5:**
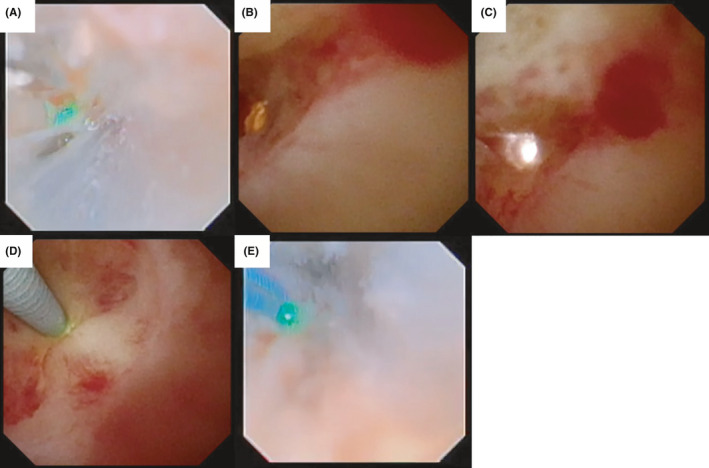
A, Ho: YAG laser incision from retrograde ureteroscopy, (B and C) lightning of retrograde ureteroscopy was observed by antegrade ureteroscopy, (D) guidewire was passed from antegrade ureteroscopy, and (E) ureteroscopic lithotripsy was performed supporting by guidewire not to forward to extra ureter

The surgical procedure was performed as follows. Under general anesthesia, the patient was placed in the Galdakao‐modified Valdivia position.^3^ We checked the ureter using a 6‐Fr rigid ureterorenoscope from the internal ureteral orifice to the stenotic lesion. A 2‐mm ureteral stone was observed at the lower ureter. This stone was removed using a stone basket. We placed a guidewire just before the stenotic lesion, and 12/14‐Fr 35‐cm ureteral access sheath (UAS) was placed under radiographic guidance. At that time, we also inserted 12/14‐Fr 35‐cm UAS from the nephrostomy to the stenotic lesion under radiographic guidance. We used a disposable flexible ureteroscope (WiScope, OUT Medical) to perform retrograde ureteroscopy and a digital flexible ureteroscope (URF‐V2, Olympus) to perform antegrade ureteroscopy. When we flushed contrast reagent, it was observed by both the retrograde and antegrade ureteroscopes; however, it did not pass the stenotic lesion, which was approximately 2 cm in length (Figure 4A). We observed the stenotic lesion using a disposable ureteroscope and a pinhole lesion observed (Figure 4B). We initially thought that this pinhole was the stenotic lesion and inserted a guidewire. However, when we inserted the guidewire, radiography demonstrated that the guidewire was beside the ureter and the guidewire was not detected by antegrade ureteroscopy (Figure 4C). Based on these findings, we suspected that the pinhole lesion had been made by the guidewire during this procedure. Although the ureter was completely obstructed, when we flushed indigo carmine liquid, it was observed by the antegrade ureteroscope (Figure 4D). When we touched the stenotic lesion with the guidewire from the antegrade ureteroscope, the movement of the ureteral mucosa at the stenotic lesion was observed by the retrograde ureteroscope. We first made a tiny incision using a 200‐µm Ho: YAG laser (SphinxJr.; settings, 0.3 J, 10 Hz) (Figure 5A). The light of the retrograde ureteroscope was observed by the antegrade ureteroscope; then, the incision was continued under observation by the antegrade ureteroscope to ensure that the incision did not proceed too far (Figure 5B and C). After dilating the ureter to allow the guidewire to pass the stenotic lesion, we inserted a guidewire from the antegrade ureteroscope (Figure 5D). This guidewire was placed from the nephrostomy and urethra as a through‐and‐through guidewire. We then re‐inserted both UASs beside this guidewire. We incised the stenotic lesion beside the guidewire; then, the residual stone was observed. We performed lithotripsy and removed the stone (Figure 5E). Because the mucosa at the incised lesion was tiny, we decided to stop the incision. 6. After confirming that a 5‐Fr ureteral catheter passed the stenotic lesion, we inserted a double J ureteral stent (Inlay Optima, BARD) and also placed a 12‐Fr nephron balloon catheter. At 1 day after surgery, the nephron balloon catheter was clamped and CT confirmed that there was no residual stone or hydronephrosis. Then, the nephron balloon catheter was removed. Three months after this procedure, we performed ureteroscopy to confirm no residural stone and exchanged to metallic ureteral stent. He was free from stenosis 3 months after this treatment. But to avoid restenosis, we planned metallic ureteral stent exchange every year.

## 3 DISCUSSION

When we identify stenotic lesions with a small lumen, a guidewire can be passed through to the upper ureter and ureteral stenting can be performed after balloon or catheter dilation.^4^, ^5^, ^6^ However, when the stenosis is so severe that contrast reagent cannot pass through to the upper ureter, endoscopic surgery cannot be performed. In such cases, uretero‐uretero anastomosis is planned to treat the stenotic lesion.^7^ However, in cases with longer stenotic lesions, this surgical approach can be difficult, especially in the presence of widespread ureteral inflammation.

We previously reported a case in which a stenotic lesion was treated with a simultaneous retrograde and antegrade approach.^2^, ^8^ In addition to radiographic assistance, visual recognition under the endoscope facilitated successful ureteral stenting. Not all institutes have multiple ureteroscopes, and to overcome this, we used a disposable ureteroscope in the present case. Recent developments have improved the resolution of disposable ureteroscopes. In the present case, a disposable ureteroscope was used for the retrograde approach. Due to the higher costs of disposable ureteroscopes, the use of a disposable ureteroscope is not indicated in all cases. When a delicate procedure is planned—such as in the present case—the use of flexible ureteroscopes with a simultaneous retrograde and antegrade approach has increased the success rate.

We reported a case of complete ureteral stenosis that was successfully managed using Ho; YAG laser incision with a simultaneous retrograde and antegrade ureteroscopic approach.

## CONFLICT OF INTEREST

The authors declare no conflicts of interest in association with the present study.

## AUTHOR CONTRIBUTIONS

TM, TK, and SK: acquired data. TM and TK: wrote the manuscript. HU: involved in supervision.

## ETHICAL APPROVAL

The patient provided their written informed consent for the publication of this study, and ethical approval was obtained.

## Data Availability

Due to ethical restrictions, the raw data underlying this paper are available upon request from the corresponding author.
